# Declared Reasons for Cessation of Breastfeeding during the First Year of Life: An Analysis Based on a Cohort Study in Northern Spain

**DOI:** 10.3390/ijerph18168414

**Published:** 2021-08-09

**Authors:** Carolina Lechosa-Muñiz, María Paz-Zulueta, Joaquín Cayón-De las Cuevas, Javier Llorca, María Jesús Cabero-Pérez

**Affiliations:** 1 Faculty of Nursing, University of Cantabria, Avda Valdecilla s/n. C.P., 39008 Santander, Spain; carolina.lechosa@unican.es; 2 Pediatrics Section, Hospital Universitario Marqués de Valdecilla, 39008 Santander, Spain; 3 IDIVAL, Grupo de Investigación en Derecho Sanitario y Bioética, GRIDES, C/Cardenal Herrera Oria s/n. C.P., 39011 Santander, Spain; 4 Faculty of Law, University of Cantabria, Avda. de los Castros s/n. C.P., 39005 Santander, Spain; joaquin.cayon@unican.es; 5 Faculty of Medicine, University of Cantabria, Avenida del Cardenal Herrera Oria 2, C.P., 39010 Santander, Spain; javier.llorca@unican.es (J.L.); mariajesus.cabero@unican.es (M.J.C.-P.); 6 CIBER Epidemiology and Public Health (CIBERESP), C.P., 28029 Madrid, Spain; 7 IDIVAL, C/Cardenal Herrera Oria s/n. C.P., 39011 Santander, Spain

**Keywords:** breastfeeding, evidence-based nursing, health promotion, women’s health, newborn

## Abstract

Background: Breastfeeding is the gold standard of infant feeding due to the many advantages it offers to both the child and the mother. Objective: To identity the main reasons for cessation of breastfeeding reported by mothers during the first year of life. Design: A prospective cohort study was conducted, recruiting 970 infants from a university hospital in Spain. The main maternal variables studied were maternal age, parity, educational level, work occupation, smoking habit, weeks of gestation at birth, birth weight, feeding type, and duration of breastfeeding. All participants were followed for one year to determinate the duration of breastfeeding and to gather reasons for abandoning breastfeeding. Results: At six months, the percentage of breastfeeding experienced a decline of 50%, and only 24.5% of these mothers maintained breastfeeding. Up to 15.8% of the mothers decided to give up exclusive breastfeeding by their own choice, whereas 15.4% did so because they suspected low milk production. Work-related causes represent the third reason of abandonment. Conclusions: Our results highlight the need to improve the health policies for the promotion, protection, and support for the initiation and maintenance of breastfeeding. In particular, our results highlight the importance of researching women’s low milk production and work-related factors, with particular emphasis on improving conciliation measures.

## 1. Introduction

Undoubtedly, breastfeeding is the gold standard for infant feeding due to the many advantages that it offers to both the infant and the mother [1,2,3,4].

This is reflected in the Global Strategy on Diet, Physical Activity, and Health (2004) and the Action Plan to Implement the Global Strategy for the Prevention and Control of Non-Communicable Diseases (2008) adopted by the World Health Organization (WHO). These strategies include the promotion of breastfeeding and complementary feeding among the interventions to reduce the common modifiable risk factors for non-communicable diseases, highlighting maternal and child nutrition as a priority area of intervention [5,6]. At present, the WHO recommendations remain essentially unchanged [7].

Likewise, the Comprehensive Implementation Plan on Maternal, Infant, and Young Child Nutrition (WHO, 2014) states that the global target for 2025 is to increase exclusive breastfeeding among infants younger than six months to at least 50% [8].

Moreover, the Academy of Medicine, the American Academy of Paediatrics (AAP), and the Spanish Association of Paediatrics (AEP) recommend exclusive breastfeeding for up six months and to continue to breastfeed together with complementary feeding for two years or more [9,10].

From the perspective of the implementation of these guidelines, according to the map published in 2016 by UNICEF, the highest rates of exclusive breastfeeding worldwide until the recommended six months are found in countries of South Asia, with rates of 60%, followed by Eastern Europe and South Africa with 57% [11,12].

In Europe, in the study carried out by Bosi et al. [13], the data from 53 European Region Member States of the WHO were analysed, observing a wide disparity in rates. Nine out of 21 countries (many countries failed to provide any data) had an initiation rate of over 50%. The lowest prevalence was observed in Bulgaria (5%) and Serbia (8%), whereas the highest rates were in Kyrgizstan (Asia), where 84% of infants started breastfeeding one hour after birth.

Under six months, the exclusive breastfeeding rates are highly variable, ranging from as little as 2% in Bulgaria and 3.7% in Poland to 56.1% in Kyrgyzstan, 54.8% in Georgia, and 52.4% in Croatia. Rates for breastfeeding at six months in Greece, Finland, and the United Kingdom only reach 1% compared to 49% in Slovakia and 44% in Hungary. At one year, the highest rate of breastfeeding was observed in Uzbekistan with 78%, followed by Turkmenistan with 6%, whereas the lowest rate was found in Greece with 6.4% and Tajikistan with 1% [13].

Regarding the current prevalence rates in Spain, these are difficult to determine considering the lack of an adequate official record of follow-up and monitoring of breastfeeding. The main source of information regarding the percentage of breastfed children is based on interviews gathered in the 2017 national health survey. This states that 74% of mothers continue to breastfeed at six weeks. Subsequently, there is a decrease at six months, at which point only 9% of women continue to breastfeed [14]. The main limitation of this survey was that it was a retrospective study that relied on maternal recall.

Having established these premises, numerous factors have been described that positively influence both the onset and the duration of breastfeeding: higher maternal education [15,16], parity [17], full term delivery [2], vaginal delivery [18,19], skin-to-skin contact between mother and child immediately after birth [20], previous experience, non-separation of the mother–child binomial, and the breastfeeding education received [21,22,23].

Therefore, the aim of our study was to identity the main reasons for cessation of breastfeeding as reported by mothers during the first year of life in Spain and to verify whether this corresponds with other studies and, finally, in the event of a divergence, to reflect on possible causes.

## 2. Materials and Methods

A prospective cohort study was carried out by recruiting 970 consecutive infants born at a university hospital in the North of Spain from 1 January 2018 to 31 August 2018. In 2020, this university hospital had 907 staffed acute-care beds and a catchment area comprising 583,904 inhabitants. Furthermore, it is a hospital of reference for two other local hospitals.

All infants born during the study period were eligible who were residents of the community regardless of their gestational age. Only those who were not going to reside in the community in the next 12 months were excluded and could not be followed-up.

### 2.1. Data Source:

Recruitment was performed at the time of maternal discharge: 48 h after delivery.

The nurse in charge provided information about the study and conducted a face-to-face interview in addition to initiating the collection of variables at the time of enrolment. These variables were obtained from the medical history of the infant and the mother. The main maternal variables studied were maternal age, parity (primiparous, multiparous), educational level (primary school, high school, vocational training, university), work occupation (student, employed and actively working, unemployed, inactive), and smoking habit (no. of cigarettes/day). The neonatal variables studied were gestational age, birth weight (normal weight, underweight, and macrosomia), feeding type (exclusive breastfeeding, breastfeeding, formula feeding), and duration of breastfeeding. The WHO definition [24] was used to categorize the type of infant feeding: exclusive breastfeeding refers to the infant receiving only breast milk (including expressed milk or from a wet nurse) and no other food or drink, not even water, except for oral rehydration salts, drops, and syrups (vitamins, minerals, and medicines); breastfeeding refers to the infant receiving breastfeeding together with formula milk; and formula feeding refers to the infant receiving formula milk exclusively.

All the participants were followed for a year to determinate the duration of breastfeeding and to gather the reason for abandonment. The latter was classified as maternal desire, low milk supply, child weaning, medical contraindication, and work-related causes.

To obtain the necessary follow-up data, health check-ups at 2, 4, 6, 9, and 12 months of age were performed by primary care nurses and paediatricians. These check-ups are performed jointly, and both professionals recorded the data in the electronic health record. During the follow-up, the investigators collected the data from the records and entered these into the study database.

### 2.2. Statistical Analysis:

Percentage distributions of infant feeding mode by time and reported reasons for breastfeeding cessation were estimated with their corresponding confidence interval (CI) at 95%.

### 2.3. Ethical-Legal Considerations:

This study was approved by the Clinical Research Ethics Committee of our region on 21 July 2017. During the hospital stay after the birth, parents were informed about the study and were requested to sign consent in order to participate. The data were pseudo-anonymized and processed confidentially according to the applicable legislation (EU) 2016/679, of 27 April 2016, on the protection of natural persons with regard to processing of personal data and the organic law 3/2018, of 5 December about personal data protection and guarantee of digital rights [25]. Each patient was identified with a unique code ensuring the confidentiality and the follow-up of medical data. Likewise, specific security measures were taken to prevent the re-identification and access of unauthorized third parties.

## 3. Results

The descriptive data of the cohort are published elsewhere [21]. The age of the mothers was 33.7 ± 5.2 years, ranging between 17 and 52 years old. A total of 53.5% were primiparous. A total of 36.2% studied at university, and about 70% were active workers (any degree of employment pre-birth). We found that 12.5% of postpartum women were smokers, with a mean consumption of 7.2 cigarettes/day. In the descriptive analysis of the employment situation of the puerperal women according to the type of infant-feeding method, no statistically significant differences were found [21].

Concerning the infants, 50.5% were male and 49.5% were female. The mean gestational age at the time of birth was 39.1 ± 1.96. A total of 93.8% were delivered at term, 4% were late preterm deliveries, and 2.2% were premature deliveries. The mean weight of infants was 3244.6 ± 572.3 g, ranging from 870 to 4840 g [21].

The prevalence of exclusive maternal breastfeeding at the time of discharge from hospital (at 48 h of life) was 53.4%. Observing the evolution of the prevalence over the 12 months studied, a considerable decrease in the rates of exclusive maternal breastfeeding occurred between months 3–4, leading to formula feeding. Thus, at six months, the percentage of maternal lactation was reduced by half, and only 24.5% of these mothers maintained breastfeeding compared to 49.8% who were exclusive formula feeding. At 12 months, only 24.6% of mothers continued to breastfeed their children (Table 1).

Of the 518 women who exclusively breastfed for some period (i.e., were exclusively breastfeeding at hospital discharge), 17.6% switched to a mixed strategy of breastmilk and formula or exclusively formula at two months; 14.1% switched to a mixed strategy of breastmilk and/or exclusively formula at four months; and 22.4% switched to a mixed strategy of breastmilk and/or exclusively formula at six months. Of the 272 women who were breastfeeding at hospital discharge, 67.3% maintained breastfeeding along with formula until six months.

The main reasons for this cessation breastfeeding are shown in Table 2. A total of 15.8% of the mothers made a personal decision to give up exclusive breastfeeding, whereas 15.4% did so because they suspected low milk supply. Regarding the mothers who chose the breastfeeding strategy, the main causes were the same: 19.5% suspected low milk supply, and 17.3% did so based on maternal choice. Work-related causes were the third reason of abandonment in both cases: 9.9% and 8.5% respectively.

## 4. Discussion

The rate of exclusive breastfeeding in infants at the time of hospital discharge was 55%, which is lower than in other communities in Spain and also very distant from the required rates by the IHAN initiative (Initiative for the Humanisation of Birth and Breastfeeding Care) [26] that recommend rates of at least 75% of exclusive breastfeeding from birth to hospital discharge

Compared to other studies, in Madrid, the ELOIN cohort reported an exclusive breastfeeding prevalence of 77.6% [27], and the CALINA study reported rates of 82.5% [28] in Aragon; the INMA cohort reported rates of 84.8% [29] in Guipuzcoa; the rates were 91.2% in Murcia [30], and in the community of Valencia, the rates for the Malam project were 81% [31].

Our study points to a drop in breastfeeding rates between three and four months. Thus, at six months, only 24.6% of women maintain exclusive breastfeeding. These findings are in line with the ELOIN study carried out in Madrid, where the exclusive breastfeeding rate was 25.4% [27]. In Aragon, the CALINA study obtained a rate of 54.3% [28]; this was higher than Guipuzcoa, where the INMA study only obtained 15.4% [29].

In the case of mothers who began exclusive breastfeeding, the main reasons for stopping breastfeeding were maternal desire (15.8%), low milk supply (15.4%), and work-related causes (9.8%). The percentages were very similar among the group of mothers who provided breastfeeding.

Our results differ from the data obtained in previous national studies in Spain regarding the reasons for the cessation of breastfeeding. In the ELOIN study [27], the most common reasons for abandoning breastfeeding were lack of milk (36%) and incorporation to work (25.9%). In the INMA study, the main reason was work related (31.1%) followed by low milk production (hypogalactia) (19.4%) [29].

The differences found could be due to various aspects. First, the maternal desire to abandon breastfeeding can be influenced by the social and cultural context in which the mother lives, considering that grandmothers are key figures in the support and intergenerational transmission of breastfeeding [30,32,33,34]. Sometimes, the maternal choice to abandon breastfeeding is due to the lack of family and social support. Further qualitative studies are needed to provide insight into maternal decisions.

The diagnosis of hypogalactia is the second important aspect highlighted in this study. Hypogalactia is a term frequently misinterpreted by mothers when they believe that their children are left hungry. Poor milk production can be caused by somatic and psychological factors. Morton proposed a three-level classification: preglandular (hormonal, nutritional, or systematic causes), glandular (primary or secondary hypoplasia), and postglandular (mother–child separation and inadequate emptying of the breast) [35]. Most women self-diagnose hypogalactia when it is a medical diagnosis. It is important for the health personnel who care for mothers and their infants to understand the true causes of hypogalactia to help mothers to manage these issues and thus avoid unwanted cessation of breastfeeding.

Further aspects include psychosocial factors as important factors that can influence milk production, triggering postglandular hypogalactia [36].

A recent study conducted in Spain by Santa Cruz et al. shows that the variables that influenced the maintenance of exclusive breastfeeding were previous decision, the belief that exclusive breastfeeding is sufficient, not offering water or liquids to the child, delaying the use of the dummy for a longer gestational period, and previous experience in the practice of exclusive breastfeeding for more than six months [37].

Considering the abandonment of breastfeeding due to work-related factors, it is important to underline the impact that legislation has on this matter. UNICEF has expressly highlighted the importance of the enactment of national laws recognizing paid maternity leave or break allowances for breastfeeding under the protection of convention 183 of the International Labour Organization in the year 2000 [12,38].

In Spain, maternity leave is 16 weeks [39] compared to other European countries; for example, in Sweden, the maternity leave is 180 days (16 months) and is shared by both the mother and the father, who receive 80% of their salary during the first 390 days of leave. In Bulgaria, mothers have a maternity leave of 410 days, receiving a full salary and with the possibility to extend leave for three years. In this case, they receive a percentage of their salary during the second year and nothing during the third year. Women in Albania, the United Kingdom, Bosnia, and Montenegro have 365 days leave (a full year). In Norway, maternity leave lasts 315 days (approximately 10 months); in Greece, the duration is 301 days (42 weeks; 10 months) compared to 294 days in Ireland (42 weeks; almost 10 months) [40]. However, it should be noted that maternity leave is clearly not the whole solution: the United Kingdom has relatively generous rights and also some of the lowest exclusive breastfeeding rates in the world.

According to the data published in the standardised OECD survey (regarding length of maternity leave, parental leave, and paid father-specific leave) used by the national breastfeeding initiative committees in 11 European countries, at six months, the countries with the highest rates of breastfeeding were Norway (71%), Sweden (61%), and Germany (57%) [41].

The incorporation of the mother back into the job market therefore puts exclusive maternal breastfeeding at risk, forcing mothers to incorporate formula milk or complementary feeding during their working hours. If mothers have a part-time job, they may be able to do this; however, if they are in full-time employment, they will most likely have to abandon breastfeeding. Numerous previously published studies have demonstrated that paid maternity leave contributes to the promotion and support of exclusive breastfeeding up to six months [38,39,40,41,42].

Studies in Brazil support the importance of increasing the period of maternity leave [42,43]. Moreover, Rimes et al. show that the performance of maternal work with maternity leave was associated with a higher outcome (APR = 1.91; 95% CI 1.32–2.78), compared with mothers who worked without maternity leave [42].

In the study published by Gramdahl et al., maternity leave during the first 24 months is associated with a longer duration of breastfeeding [44]. Chai et al., with a sample of 1,000,753 children under five years of age from 38 countries, showed that a 1-month increase in the legislated duration of paid maternity leave was associated with a 7.4 percentage-point increase (95%  CI 3.2 to 11.7) in the prevalence of early initiation of breastfeeding, a 5.9 percentage-point increase (95%  CI 2.0 to 9.8) in the prevalence of exclusive breastfeeding, and a 2.2-month increase (95%  CI 1.1 to 3.4) in breastfeeding duration [45]. Employed women who received 12 or more weeks of paid maternity leave were more likely to initiate breastfeeding and be breastfeeding their child at six months than those without paid leave [46]. In the study by Smith et al. among Irish mothers, a shorter duration of breastfeeding was significantly associated with mothers whose maternity leave was between seven and 12 months (adjusted OR = 2.76, 95% CI 1.51–5.05) [47].

Steurer highlighted the importance of longer maternity leave but also stressed the importance of facilitators for breastfeeding maintenance as well as adequate time and space for expressing breastmilk once the mother has returned to the workplace [48].

In the USA, health policies have been implemented to support and protect breastfeeding with reimbursed licences [49]. In the study by Huang et al. evaluating the implementation of the programme, they found an increase of 3–5 percentage points for exclusive breastfeeding and an increase of 10–20 percentage points for breastfeeding [50].

## 5. Conclusions

Our results show the need to improve health policies for the promotion, protection, and support for the initiation and continuation of breastfeeding. Further qualitative studies are needed to delve into maternal decisions. Additional lines of research are also required to address women’s low milk production.

In particular, our results show the importance of work factors, with particular emphasis on improving conciliation measures to enable mothers to maintain exclusive maternal breastfeeding up to six months if they so desire and also be able to continue with supplementary feeding up to two years or more.

Nonetheless, further research into the causes of breastfeeding cessation is recommended, as this is essential to design programs to help mothers to continue breastfeeding if they wish to do so.

### 5.1. Limitations

In studies based on secondary information (records), one of the main limitations is the poorer quality of the information. This may be due to a lack of agreement in information provided through different records or to an insufficient completion of the medical records required for the study. To minimize these biases, we purposely chose the variables that were collected in a more homogeneous, systematic, and objective manner in the electronic medical records. Likewise, prior to the definitive inclusion of the variables, an assessment was made to determine the concordance between the data accessed from different sources.

Due to this limitation, we have not been able to systematically collect and analyse certain variables of interest in our study: details about the specific context in which our sample of mothers are living, prevalence of the use of breast pumps in Spain, or the percentage of work dropout once women start childbearing.

### 5.2. Strengths

The data in this study were analysed by a multi-professional team with different academic and professional backgrounds: paediatricians, nurses, researchers, an epidemiologist, and a lawyer. The diverse perspectives enrich the interpretation of the data, the analyses, and the discussion of the findings.

## Figures and Tables

**Table 1:** Prevalence of breastfeeding and its evolution over 12 months.

Moment	Type of Feeding	n	%	95% CI	
Hospital discharge	Exclusive breastfeeding	518	53.40	50.21	56.59
	Breastfeeding	272	28.04	25.16	30.92
	Formula feeding	174	17.94	15.47	20.40
	Human milk donated	6	0.62	0.07	1.16
2 months	Exclusive breastfeeding	427	44.02	40.89	47.24
	Breastfeeding	183	18.87	16.37	21.40
	Formula feeding	299	30.82	27.90	33.82
	Missing data	60	6.29	4.62	7.76
4 months	Exclusive breastfeeding	354	36.49	33.45	39.62
	Breastfeeding	164	16.91	14.51	19.34
	Formula feeding	387	39.90	36.80	43.07
	Missing data	64	6.70	4.99	8.22
6 months	Exclusive breastfeeding	238	24.54	21.80	27.32
	Breastfeeding	183	18.87	16.37	21.40
	Formula feeding	483	49.79	46.65	53.05
	Missing data	65	6.80	5.08	8.34
9 months	Exclusive breastfeeding	0	0	-	-
	Breastfeeding	318	32.78	29.81	35.83
	Formula feeding	573	59.07	55.99	62.28
	Missing data	77	8.14	6.19	9.70
12 months	Exclusive breastfeeding	0	0	-	-
	Breastfeeding	239	24.64	21.90	27.43
	Formula feeding	642	66.19	63.23	69.28
	Missing data	88	9.18	7.22	10.94

Exclusive breastfeeding, breast milk only; Breastfeeding, breast milk and formula milk; Formula feeding, formula milk only [24].

**Table 2:** Main reasons for cessation of breastfeeding.

	Exclusive Breastfeeding				Breastfeeding			
	n = 518	%	95% CI		n = 272	%	95% CI	
Reasons for cessation								
Maternal desire	82	15.83	12.59	19.07	47	17.28	12.60	21.96
Low milk supply	80	15.44	12.24	18.65	53	19.49	14.60	24.38
Work-related causes	51	9.85	7.18	12.51	23	8.46	4.97	11.95
Child weaning	26	5.02	3.04	7.00	15	5,51	2.62	8.41
Contraindication	4	0.77	0.21	1.97	1	0.37	0.01	2.03
Missing	71	13.71	10.65	16.77	44	16.18	11.62	20.74

Exclusive breastfeeding, breast milk only; Breastfeeding, breast milk and formula milk [24].

## Data Availability

The data presented in this study are available on request from the corresponding author. The data are not publicly available due to patients’ privacy.

## References

[1] Gertosio C., Meazza C., Pagani S., Bozzola M. (2016). Breastfeeding and its gamut of benefits. Minerva Pediatr..

[2] World Health Organization (WHO) Short-Term Effects of Breastfeeding: A Systematic Review on the Benefits of Breastfeeding on Diarrhoea and Pneumonia Mortality. https://apps.who.int/iris/bitstream/handle/10665/95585/9789241506120_eng.pdf?sequence=1&isAllowed=y.

[3] Horta B.L., Loret de Mola C., Victora C.G. (2015). Long-Term Consequences of Breastfeeding on Cholesterol, Obesity, Systolic Blood Pressure and Type 2 Diabetes: A Systematic Review and Meta-Analysis. Acta Paediatr..

[4] Islami F., Liu Y., Jemal A., Zhou J., Weiderpass E., Colditz G., Boffetta P., Weiss M. (2015). Breastfeeding and breast cancer risk by receptor status–A systematic review and meta-analysis. Ann. Oncol..

[5] World Health Organization (2004). Global Strategy for Infant and Young Child Feeding (WHA57.17). https://apps.who.int/iris/bitstream/handle/10665/42590/9241562218.pdf?sequence=1.

[6] World Health Organization (2008). Prevention and Control of Noncommunicable Diseases: Implementation of the Global Strategy (WHA61.14)). https://www.who.int/ncds/governance/2008-resolution-which-endorsed-GAP.pdf?ua=1.

[7] World Health Organization Promoting Proper Feeding for Infants and Young Children. https://apps.who.int/nutrition/topics/infantfeeding/en/index.html.

[8] World Health Organization (2014). Comprehensive Implementation Plan on Maternal, Infant and Young Child Nutrition (WHO/NMH/NHD/14.1). https://apps.who.int/iris/bitstream/handle/10665/113048/WHO_NMH_NHD_14.1_eng.pdf?sequence=1&isAllowed=y.

[9] Holmes A.V., McLeod A.Y., Bunik M. (2013). ABM Clinical Protocol #5: Peripartum breastfeeding management for the healthy mother and infant at term, revision 2013. Breastfeed Med..

[10] Asociación Española de Pediatría (AEP) Recomendaciones Sobre Lactancia Materna del Comité de Lactancia Materna de la Asociación Española de Pediatría [Recommendations on Breastfeeding by the Committee of Maternal Breastfeeding of the Spanish Association of Pediatrics]. https://www.aeped.es/comite-lactancia-materna/documentos/recomendaciones-sobre-lactancia-maternacomite-lactancia-materna.

[11] Proyecto de la UE Sobre la Promoción de la Lactancia en Europa Protección, Promoción y Apoyo de la Lactancia en Europa: Plan Estratégico Para la Acción. Comisión Europea, Dirección Pública de Salud y Control de Riesgos. https://www.aeped.es/sites/default/files/5-europe_a_blueprint_for_action.pdf.

[12] UNICEF (2016). From the First Making the Case for from the First. http://data.unicef.org/resources/first-hour-life-new-report-breastfeedingpractices/.

[13] Bagci Bosi A.T., Eriksen K.G., Sobko T., Wijnhoven T.M., Breda J. (2016). Breastfeeding practices and policies in WHO European Region Member States. Public Health Nutr..

[14] Ministerio de Sanidad, Servicios Sociales e Igualdad Instituto Nacional de Estadística. http://www.ine.es/ss/Satellite?L=es_ES&c=INESeccion_C&cid=1259926698156&p=1254735110672&pagename=ProductosYServicios%2FPYSLayout.

[15] Craighead D.V., Elswick R.K. (2014). The influence of early-term birth on NICU admission, length of stay, and breastfeeding initiation and duration. JOGNN.

[16] Busck-Rasmussen M., Villadsen S.F., Norsker F.N., Mortensen L., Andersen A.M. (2014). Breastfeeding practices in relation to country of origin among women living in Denmark: A population-based study. Matern Child Health J..

[17] Griffiths L.J., Tate A.R., Dezateux C. (2005). The millennium cohort study child health group: The contribution of parental and community ethnicity to breastfeeding practices: Evidence from the millennium cohort study. Int. J. Epidemiol..

[18] Apostolakis-Kyrus K., Valentine C., DeFranco E. (2013). Factors associated with breastfeeding initiation in adolescent mothers. J Pediatr..

[19] Häggkvist A.P., Brantseter A.L., Grjibovski A.M., Helsing E., Meltzer H.M., Haugen M. (2010). Prevalence of breast-feeding in the norwegian mother and child cohort study and health service-related correlates of cessation of full breast-feeding. Public Health Nutr..

[20] Leung G.M., Ho L.M., Lam T.H. (2002). Maternal, paternal and environmental tobacco smoking and breastfeeding. Paediatr. Perinat. Epidemiol..

[21] Lechosa Muñiz C., Paz-Zulueta M., Del Río E.C., Sota S.M., Sáez de Adana M., Pérez M.M., Cabero Pérez M.J. (2019). Impact of maternal smoking on the onset of breastfeeding versus formula feeding: A cross-sectional study. Int. J. Environ. Res. Public Health.

[22] Bramson L., Lee J.W., Moore E., Montgomery S., Neish C., Bahjri K., Melcher C.L. (2010). Effect of early skin-to-skin mother-infant contact during the first 3 hours following birth on exclusive breastfeeding during the maternity hospital stay. J. Hum. Lact..

[23] Cohen S.S., Alexander D.D., Krebs N.F., Young B.E., Cabana M.D., Erdmann P., Hays N.P., Bezold C.P., Levin-Sparenberg E., Turini M. (2018). Factors associated with breastfeeding initiation and continuation: A Meta-Analysis. J. Pediatr..

[24] World Health Organization Indicators for Assessing Infant and Young Child Feeding Practices; Part 1: Definitions. https://apps.who.int/iris/bitstream/handle/10665/43895/9789241596664_eng.pdf?sequence=1.

[25] Ley Orgánica 3/2018, de 5 de Diciembre, de Protección de Datos Personales y Garantía de los Derechos Digitales. https://www.boe.es/buscar/doc.php?id=BOE-A-2018-16673.

[26] iHan Iniciativa para la Humanización de la Asistencia al Nacimiento y la Lactancia. https://www.ihan.es/.

[27] Ramiro González M.D., Ortiz Marrón H., Arana Cañedo-Argüelles C., Esparza Olcina M.J., Cortés Rico O., Terol Claramonte M., Ordobás Gavín M. (2018). Prevalencia de la Lactancia Materna y Factores Asociados con el Inicio y la Duración de la Lactancia Materna Exclusiva en la Comunidad de Madrid Entre los Participantes en el Estudio ELOIN. [Prevalence of Breastfeeding and Factors Associated with the Initiation and Duration of Exclusive Breastfeeding in the Community of Madrid among Partipants in the ELOIN Study]. An Pediatr..

[28] Cuadrón Andrés L., Samper Villagrasa M.P., Álvarez Sauras M.L., Lasarte Velillas J.J., Rodríguez Martínez G. (2013). Prevalencia de la lactancia materna durante el primer año de vida en Aragón. Estudio CALINA. An. Pediatr..

[29] Oribe M., Lertxundi A., Basterrechea M., Begiristain H., Santa Marina L., Villar M., Dorronsoro M., Amiano P., Ibarluzea J. (2015). Prevalencia y factores asociados con la duración de la lactancia materna exclusiva durante los 6 primeros meses en la cohorte INMA de Guipúzcoa. Gac Sanit..

[30] Ortega García J.A., Pastor Torres E., Martínez Lorente I., Bosch Giménez V., Quesada López J.J., Hernán-dez Ramón F., Alcaráz Quiñonero M., Llamas del Castillo M.M., Torres Cantero A.M., García de León González R. (2008). Proyecto Malama en la región de Murcia (España): Medio ambiente y lactancia materna. An. Pediatr..

[31] Rius J.M., Ortuño J., Rivas C., Maravall M., Calzado M.A., López A., Aguar M., Vento M. (2014). Factores asociados al abandono precoz de la lactancia materna en una región del este de España [Factors associated with early weaning in a Spanish region]. An Pediatr..

[32] Angelo B.H.B., Pontes C.M., Sette G.C.S., Leal L.P. (2020). Knowledge, attitudes and practices of grandmothers related to breastfeeding: A meta-synthesis. Rev. Lat. Am. Enfermagem..

[33] Rodriguez Vazquez R., Losa-Iglesias M.E., Corral-Liria I., Jiménez-Fernández R., Becerro-de-Bengoa-Vallejo R. (2017). Attitudes and Expectations in the Intergenerational Transmission of Breastfeeding: A Phenomenological Study. J. Hum. Lact..

[34] Negin J., Coffman J., Vizintin P., Raynes-Greenow C. (2016). The influence of grandmothers on breastfeeding rates: A systematic review. BMC Pregnancy Childbirth.

[35] Comité de lactancia materna de la Asociación Española de Pediatría (2008). Manual de Lactancia Materna. De la Teoría a la Práctica.

[36] Machado M.C., Assis K.F., Oliveira Fde C., Ribeiro A.Q., Araújo R.M., Cury A.F., Priore S.E., Franceschini Sdo C. (2014). Determinants of the exclusive breastfeeding abandonment: Psychosocial factors. Rev. Saude Publica.

[37] Santacruz-Salas E., Segura-Fragoso A., Pozuelo-Carrascosa D.P., Cobo-Cuenca A.I., Carmona-Torres J.M., Laredo-Aguilera J.A. (2021). Maintenance of Maternal Breastfeeding up to 6 Months: Predictive Models. J. Pers. Med..

[38] International Labour Organization Maternity Protection Convention, 2000 (No. 183). https://www.ilo.org/dyn/normlex/en/f?p=NORMLvEXPUB:12100:0::NO::P12100_ILO_CODE:C183.

[39] Real Decreto-ley 6/2019, de 1 de Marzo, de Medidas Urgentes Para Garantía de la Igualdad de Trato y de Oportunidades Entre Mujeres y Hombres en el Empleo y la Ocupación. https://www.boe.es/eli/es/rdl/2019/03/01/6/con.

[40] Maternity and Paternity at Work: Where do Mothers Get More Leave? [Internet]. http://www.ilo.org/global/about-the-ilo/multimedia/maps-and-charts/WCMS_241698/lang--es/index.htm.

[41] OECD Gender Initiative Length of Maternity Leave, Parental Leave and Paid Father-Specific Leave. http://www.oecd.org/gender/data/length-of-maternity-leave-parental-leave-and-paid-father-specific-leave.htm.

[42] Rimes K.A., Oliveira M.I.C., Boccolini C.S. (2019). Maternity leave and exclusive breastfeeding. Rev. Saude Publica..

[43] Monteiro F.R., Buccini G.D.S., Venâncio S.I., da Costa T.H.M. (2017). Influence of maternity leave on exclusive breastfeeding. J. Pediatr..

[44] Grandahl M., Stern J., Funkquist E.L. (2020). Longer shared parental leave is associated with longer duration of breastfeeding: A cross-sectional study among Swedish mothers and their partners. BMC Pediatr..

[45] Chai Y., Nandi A., Heymann J. (2018). Does extending the duration of legislated paid maternity leave improve breastfeeding practices? Evidence from 38 low-income and middle-income countries. BMJ Glob. Health.

[46] Mirkovic K.R., Perrine C.G., Scanlon K.S. (2016). Paid Maternity Leave and Breastfeeding Outcomes. Birth.

[47] Smith H.A., O’B Hourihane J., Kenny L.C., Kiely M., Murray D.M., Leahy-Warren P. (2015). Early life factors associated with the exclusivity and duration of breast feeding in an Irish birth cohort study. Midwifery.

[48] Steurer L.M. (2017). Maternity Leave Length and Workplace Policies’ Impact on the Sustainment of Breastfeeding: Global Perspectives. Public Health Nurs..

[49] (2011). Centers for Disease C: The Surgeon General’s Call to Action to Support Breastfeeding. https://www.cdc.gov/breastfeeding/resources/calltoaction.htm.

[50] Huang R., Yang M. (2015). Paid maternity leave and breastfeeding practice before and after California’s implementation of the nation’s first paid family leave program. Econ. Hum. Biol..

